# Tapping Into Multiple Data “Springs” to Strengthen Policy Streams: A Guide to the Types of Data Needed to Formulate Local Retail Tobacco Control Policy

**DOI:** 10.5888/pcd16.180282

**Published:** 2019-04-04

**Authors:** Allison E. Myers, Kathleen Knocke, Jennifer Leeman

**Affiliations:** 1 Counter Tools, Inc, Carrboro, North Carolina; 2 Department of Health Behavior, Gillings School of Global Public Health, University of North Carolina, Chapel Hill, North Carolina; 3 Department of Health Policy and Management, Gillings School of Global Public Health, University of North Carolina, Chapel Hill, North Carolina; 4 School of Nursing, University of North Carolina, Chapel Hill, North Carolina

## Abstract

In 2015, the tobacco industry spent $8.24 billion to market tobacco products in convenience stores, supermarkets, pharmacies, and other retail or point-of-sale settings. Community tobacco control partnerships have numerous evidence-based policies (eg, tobacco retailer licensing and compliance, tobacco-free–school buffer zones, eliminating price discounts) to counter point-of-sale tobacco marketing. However, deciding which point-of-sale policies to implement — and when and in what order to implement them — is challenging. The objective of this article was to describe tools and other resources that local-level tobacco use prevention and control leaders can use to assemble the data they need to formulate point-of-sale tobacco policies that fit the needs of their communities, have potential for public health impact, and are feasible in the local policy environment. We were guided by Kingdon’s theory of policy change, which contends that windows of policy opportunity open when 3 streams align: a clear problem, a solution to the problem, and the political will to work for change. Community partnerships can draw on 7 data “springs” to activate Kingdon’s streams: 1) epidemiologic and surveillance data, 2) macro retail environment data, 3) micro retail environment data, 4) the current policy context, 5) local legal feasibility of policy options, 6) the potential for public health impact, and 7) political will.

**Summary**

**What is already known on this topic?**

A menu of policy options exist to combat the influence of tobacco industry marketing in retail stores or at the point of sale.

**What is added by this report?**

This report offers a guide to public health practitioners and community coalitions as they decide which retail tobacco policies to pursue, and where and when.

**What are the implications for public health practice?**

The guidance here may help communities formulate point-of-sale tobacco policies that fit their needs, have potential for public health impact, and are feasible in the local policy environment.

## Introduction

The tobacco industry spends $8.24 billion annually marketing tobacco products in convenience stores, supermarkets, pharmacies, and other retail or point-of-sale (POS) settings (1). Exposure to tobacco marketing (eg, product displays, advertisements, price discounts) increases the likelihood that youth will start using tobacco and impedes users’ attempts to quit (2–4). In communities that have a high density of tobacco retail outlets, often measured as the spatial concentration of retail outlets in a geographic area, residents are exposed to more POS tobacco marketing and have higher tobacco use rates (5,6). Retail outlet density is disproportionately high in lower-income and African American communities, and this disproportionate density likely contributes to disparities in tobacco use and tobacco-related morbidity and mortality (7,8). Retail outlets that are close to schools increase the exposure to children and adolescents, who are more susceptible to the effects of tobacco marketing than adults (4,9).

Policies to reduce retail tobacco marketing include laws, ordinances, or resolutions to regulate tobacco product sales, placement, advertisements, prices, and price promotions. They also include licensing laws and zoning regulations to reduce the number and density of retail outlets or prohibit tobacco retail outlets near schools (10–13). The potential for communities to reduce retail marketing is evidenced by recent policy wins to reduce tobacco retailer density in San Francisco and Philadelphia, restrict menthol-flavored and candy-flavored tobacco sales in Minneapolis and Oakland, and raise the age of sale to 21 years in hundreds of US localities (14). Still, little is known about how best to support community efforts to promote retail tobacco policy change (15,16). One challenge faced by community partnerships is understanding which retail tobacco control policy options to pursue, when to pursue them, and in what order they should be pursued in their geographic area of interest (eg, city, county).

Although the evidence base for POS tobacco policies is still emerging, numerous resources exist to help communities select the most promising POS policy options (13,17–20). Given the variation in the range of POS policy options and their potential impact, communities need to draw on a variety of data to select the most promising options. In this article, we built on Kingdon’s theory of policy change (21) to identify the types of data that are essential to community decision making.

In his multiple streams theory, Kingdon posited that policy change is most likely to occur when 3 policy “streams” align: 1) a local problem is documented, 2) a policy solution is available, and 3) the political will is present to work toward a solution to the problem (21). This article describes 7 data “springs” that communities can draw on to select and promote policies in ways that align with Kingdon’s 3 policy streams and create windows of opportunity for new policy enactment. The objective of this article was to 1) describe the 7 data springs, 2) recommend existing sources and data collection and analysis tools for each spring, and 3) suggest how community partnerships might apply these data to strategically select and promote policy solutions to counter retail tobacco marketing.

## The 7 Data Springs

Seven data springs can contribute to community-level efforts to align with Kingdon’s 3 streams and thereby open windows of opportunity for retail tobacco marketing policy change (Figure). The first 3 springs are 1) epidemiologic/surveillance data, 2) macro retail environment data, and 3) micro retail environment data, each contributing to Kingdon’s first policy stream — documenting the local problem. The next 3 springs are 4) the current policy context, 5) the local legal feasibility of policy options, and 6) the potential for public health impact, and they relate to Kingdon’s second stream — form a policy solution. The final spring — political will — contributes to Kingdon’s final stream. Below we describe each of the data springs and recommend existing data sources and tools for collecting and analyzing information for each spring (Table).

**Figure:**
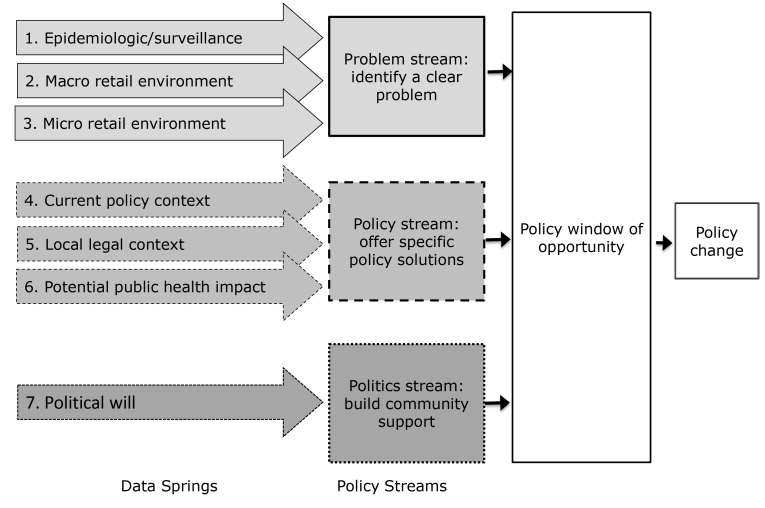
A conceptual framework indicating data springs and policy streams that merge to create policy change.

**Table:** The 7 Data Springs of Information for Formulating Local Retail Tobacco Control Policy as They Align With Kingdon’s 3 Policy Streams^a^

Policy Stream/Data Spring	Definition of Data Spring	Sources of Available Information
**Policy stream 1: Local problem**		
1. Epidemiologic and surveillance data	Tobacco use, death and disability rates among and between disparate population groups; economic and productivity losses from tobacco use	State Tobacco Activities Tracking and Evaluation (STATE) System from CDC (www.cdc.gov/STATESystem/); Campaign for Tobacco-Free Kids (www.tobaccofreekids.org); Toll of Tobacco in the United States fact sheets (www.tobaccofreekids.org/problem/toll-us); American Lung Association State of Tobacco Control, State Grades (www.lung.org/our-initiatives/tobacco/reports-resources/sotc/); County Health Rankings and Roadmaps National Youth (www.countyhealthrankings.org/); Adult Tobacco Surveys from CDC (www.cdc.gov/tobacco/data_statistics/surveys/nats/index.htm); 500 Cities Project from CDC, CDC Foundation, and Robert Wood Johnson Foundation (www.cdc.gov/500cities/)
2. Macro retail environment	Number, location, and density of tobacco outlets; proximity of tobacco retail outlets to youth-serving venues such as parks and schools; relationship between density and proximity and demographic characteristics (eg, percentage of minority race/ethnicity or percentage of low-income populations)	Tobacco retail outlet lists can be obtained from tobacco retailer licensing lists; tax tracking at departments of revenue; policy compliance checklists from the Synar Program (www.samhsa.gov/synar) or FDA enforcement; business lists from vendors; internet search engines; sale of alcoholic beverages for off-premise consumption (proxy); PhenX measures (www.phenxtoolkit.org): neighborhood-level racial/ethnic composition, youth cigarette purchase behaviors and experiences, media use, compliance with cigarette packaging and labeling policies, tobacco retailer density/proximity to administrative neighborhoods, known residences and schools
		Demographic data can be obtained from US Census or American Community Survey (www.census.gov/programs-surveys/acs/); park data from municipalities, universities, states, or Esri USA Parks (www.esri.com/software/arcgis/arcgis-for-parks-gardens); school data from US Department of Education (www2.ed.gov/rschstat/landing.jhtml)
3. Micro retail environment	Availability and marketing of tobacco products	Store assessments via Standardized Tobacco Assessment for Retail Settings (STARS) and Standardized Tobacco Assessment for Retail Settings: Vape Shops (vSTARS) from http://countertobacco.org/resources-tools/store-assessment-tools/; PhenX measures (www.phenxtoolkit.org): self-reported tobacco product paid price and self-reported tobacco product purchase location
**Policy stream 2: Policy solution**		
4. Policy context	Amount of tobacco excise tax; strength of clean indoor air law; security and amount of tobacco control funding	American Lung Association State Legislated Actions on Tobacco Issues Database (SLATI) (www.lungusa2.org/slati/states.php); State Tobacco Activities Tracking and Evaluation (STATE) System from CDC (www.cdc.gov/STATESystem/); Association for Non-smokers’ Rights Foundation database (www.no-smoke.org); Tobacconomics.org website
5. Legal feasibility	Likelihood of court challenge; case law; level of preemption; status of local tobacco retailer licensing policies	Point of Sale Strategies, a Tobacco Control Guide from Tobacco Control Legal Consortium and Center for Public Health Systems Science at Washington University in St Louis (www.publichealthlawcenter.org/sites/default/files/resources/tclc-guide-pos-policy-WashU-2014.pdf); legal consultation with Public Health Law Center (www.publichealthlawcenter.org); ChangeLab Solutions (www.changelabsolutions.org); Public Health Advocacy Institute (www.phaionline.org/); Legal Resource Center for Public Health Policy (www.law.umaryland.edu/programs/publichealth/) or other legal technical assistance provider; legal arguments found in 2016 Surgeon General’s report (www.surgeongeneral.gov/library/reports/index.html)
6. Potential for public health impact	Expected impact of POS tobacco policy solutions on tobacco sales and marketing; tobacco retail outlet number, density, proximity; tobacco use and cessation rates; tobacco-related health disparities	CDC Best Practices for Tobacco Control Programs (www.cdc.gov/tobacco/stateandcommunity/best_practices/index.htm); Institute of Medicine Public Health Implications of Raising the Minimum Age of Legal Access to Tobacco Products (www.nationalacademies.org/hmd/~/media/Files/Report%20Files/2015/TobaccoMinAge/tobacco_minimum_age_report_brief.pdf); evaluations of implemented POS tobacco policies; peer-reviewed research studies (eg, SimSmoke [https://resources.cisnet.cancer.gov/registry/packages/simsmoke-georgetown/] or TobaccoTown models [https://cphss.wustl.edu/Products/ProductsDocuments/ASPiRE_Luke_2016_ASPiREMeetingSTL_ComputationalModelingforStudyingRetailDensity.pdf]); PhenX ToolKit for Tobacco Regulatory Science (https://original-phenxtoolkit.rti.org); National Cancer Institute State and Community Tobacco Control Research Initiative (https://cancercontrol.cancer.gov/brp/tcrb/sctc.html); evidence summaries published at http://countertobacco.org/resources-tools/evidence-summaries/
**Policy stream 3: Political will**		
7. Political will	Policy decision maker and public support for POS tobacco policy solutions; community interest for POS action	Public opinion polling (http://countertobacco.org/resources-tools/public-opinion-surveys/); 1:1 conversations with policy decision makers. Building community capacity to refute tobacco industry arguments: CounterTobacco.org and Campaign for Tobacco-Free Kids (www.tobaccofreekids.org)

Abbreviations: CDC, Centers for Disease Control and Prevention; FDA, Food and Drug Administration; POS, point of sale.

a In his multiple streams theory, Kingdon posited that policy change is most likely to occur when 3 policy streams align: 1) a local problem is documented, 2) a policy solution is available, and 3) the political will is present to work toward a solution to the problem (21).

### Data spring 1: Epidemiologic and surveillance data

Epidemiologic and surveillance data include rates of tobacco product use and tobacco-related disability and death and the distribution of those rates across population groups in a geographic area of interest. Frequently, a geographic area of interest is a city, a county, or a group of counties. However, the focus may also be on nonincorporated areas of a county. Commonly used sources of state data include the Behavioral Risk Factor Surveillance System (BRFSS), the State Tobacco Activities Tracking and Evaluation (STATE) System, the Toll of Tobacco in the United States fact sheets available from the Campaign for Tobacco-Free Kids, and the Youth Risk Behavior Survey (YRBS). Local surveillance data are available from state health department websites; additional local tobacco indicators are available from programs such as the BRFSS’ Selected Metropolitan Area Risk Trends (SMART) project, the County Health Rankings, the 500 Cities Project, and the Big Cities Health Coalition. Key metrics are rates of tobacco product use among adults and adolescents according to such characteristics as sex; race/ethnicity; age; income; education; personal status, such as identifying as lesbian, gay, bisexual, or transgender or living with mental illness; or other characteristics that increase risk for tobacco use. Community partnerships should identify the populations who have the highest rates of tobacco use; this information can be used to educate community members and policy decision makers and to identify policy solutions with potential to eliminate disparities.

### Data spring 2: Macro retail environment

Data on the macro retail environment include the number and types of tobacco retail outlets in a geographic area of interest, where they are located, and how they are clustered. Tobacco retail outlets are defined as any brick-and-mortar location that sells any type of tobacco product, including conventional combustible cigarettes and electronic devices and products with or without menthol, fruit, or other flavors. Common tobacco retail outlet types are convenience stores with or without gas stations, grocery stores, mass merchandisers, pharmacies, and tobacco-only specialty shops.

Characterization of the macro retail environment begins with obtaining or creating a list of tobacco retail outlet locations. Ideal options are to obtain a tobacco retailer licensing list or create a list by “ground truthing” or canvassing an area to identify every store that sells tobacco (22). Where licensing lists are not available, substitute lists can be derived from free or purchasable business lists from internet searches of vendors (eg, Dun and Bradstreet, Reference USA); lists of stores eligible for the policy compliance and enforcement activities of the Synar Program (www.samhsa.gov/synar) or the Food and Drug Administration; or lists of stores that sell alcohol for off-premise consumption (a proxy for a tobacco retail outlet). Tobacco tax tracking through departments of revenue may also serve as a suitable substitute list; however, only products taxed at the state level will appear on this list. Details about the sensitivity and specificity of various lists, sampling strategies, and list-cleaning protocols are published elsewhere (22,23).

The location of each retailer can then be geocoded or otherwise plotted on a map according to latitude and longitude. Beyond the number, location, and type of outlets, summary information needs to specify features or problems in the macro retail environment that are amenable to change through policy. Examples of these features include the number of retail outlets that are close (≤1,000 feet) to institutions that serve children, such as schools, parks, or child care centers, and the relationship between the number and density of tobacco retail outlets, often measured as the spatial concentration of retail outlets in a geographic area (eg, neighborhood, school zone). These analyses give a visual representation of the retail tobacco landscape and the health inequities that may be changeable with policy solutions; for example, a 1,000-feet tobacco-sales–free buffer around schools has potential to reverse disparities in density by race/ethnicity (8).

### Data spring 3: Micro retail environment

A third data type includes features of the micro store environment, defined as information about the availability, price, placement, and promotion of tobacco products. Store assessments are the primary method for collecting these data and involve public health staff, community members, and other stakeholders who systematically collect observational data inside and outside retail stores. A guide to conducting store assessments is available (24). Store assessment resources are available at CounterTobacco.org, including the Standardized Tobacco Assessment for Retail Settings (STARS), with additional modules for vaping devices and electronic products (25,26). Data may include the percentage of stores in a geographic area of interest that offer tobacco price promotions (eg, cents off or multipack offers that undermine tobacco excise tax policies and promote consumption). Store assessments can be used to identify localized tobacco industry targeting (eg, lower than average prices on smokeless tobacco products in rural and low-income areas, or higher prevalence of sales of individually packaged candy-flavored or fruit-flavored tobacco products in neighborhoods with a high proportion of children). For example, in a study conducted in Milwaukee, Wisconsin, the STARS tool showed harmful POS disparities in zip codes where the population was predominantly African American and Hispanic as well as high levels of outdoor pricing and price promotion for menthol products (27). Matching localized industry targeting to policy provisions according to the STARS *Policy Crosswalk* (28) is an important element of formulating policy strategy.

### Data spring 4: Current tobacco control policy context

This fourth data type involves cataloging state policies and characteristics to assess readiness to enact local tobacco retail marketing policies. The State of Tobacco Control state grades (American Lung Association), the State Tobacco Activities Tracking and Evaluation System (STATE), and the State Legislated Actions on Tobacco Issues (SLATI) database each offer state snapshots of tobacco control progress on established Best Practice strategies from the Centers for Disease Control and Prevention (CDC) (29). When users retrieve these data, they should first note the presence and amounts of state tax on combustible and noncombustible tobacco products and the date of the last tax increase (30). Next, users should note the strength of smoke-free rules and policies by identifying the percentage of households and people who are protected by smoke-free rules. Third, users should clarify the level of tobacco control program funding: what percentage of CDC-recommended funds are being allocated to comprehensive tobacco control programming? These data help to gauge a state’s or a locality’s readiness to work on enacting POS tobacco policies: readiness may be greatest when a state has in place robust tobacco taxes, strong smoke-free rules, and secure program funding (31). In states with lower levels of readiness, tobacco control partnerships may need to focus on raising awareness of the tobacco retail marketing problem as one component of an overall strategy to prioritize the need for continued work in tobacco control more broadly. In other words, they may need to collect data on the problem to demonstrate to decision makers exactly where and how “tobacco is not finished.” In many states, participatory community-level documentation of the retail marketing problem will serve as an energizer to the broader tobacco control movement.

### Data spring 5: Local legal feasibility of policy options

A fifth data type involves understanding which retail policy solutions are feasible for the geographic area of interest, information that can be acquired through consultation with a legal technical assistance provider who specializes in tobacco control policy. A public health attorney can provide legal technical assistance and can clarify whether express or implied preemption is a barrier to progress, meaning whether existing state law precludes localities from passing certain types of tobacco control policies. A public health attorney can clarify details of existing tobacco retailer licensing policies and the extent to which they are being enforced, how they might serve as a foundation for future efforts, and the relative potential for legal success related to various policy solutions in the geographic area of interest. Public health attorneys can provide assistance in navigating policy interventions that may conflict with the First Amendment by restricting free speech through advertising or commercial speech. Contact information for legal technical assistance providers is available (Table).

### Data spring 6: Potential for public health impact

Data types essential to formulating solutions are research findings on the potential of policies to reduce exposure to tobacco marketing and/or reduce the prevalence of tobacco use at the population level. In formulating a policy solution, the potential for public health impact needs to be assessed along with data from the other streams. Data from streams 1, 2, and 3 can be applied to compare each policy option’s potential for impact given the local problem. For example, the impact of retailer licensing policies to reduce the number and density of outlets will be greatest in areas that have problems with high retailer density in low-income neighborhoods or many retailers in close proximity to schools. Data from spring 5 may influence a community partnership’s decision to select a policy option that may have less evidence in support of its effectiveness than other options but has strong legal feasibility and therefore may provide an early win that the partnership can build on to create the political will needed for future efforts to enact policy solutions with potentially greater impact.

Data on policy effectiveness is emerging. Raising the price of tobacco products is the most effective way to reduce overall consumption; this principle applies to both gold-standard tax-based approaches (eg, an excise tax) and newer nontax approaches that restrict tobacco industry strategies to minimize price (32) such as prohibiting price discounts, enacting minimum price laws, or prohibiting coupon redemption (33,34). Strategies that limit the number, type, location, and density of outlets also have good evidence in support of their effectiveness at reducing exposure to tobacco marketing and smoking rates (8,35). Policies that restrict or remove tobacco product displays in retail outlets (eg, display bans) have resulted in reductions among consumers in noticing or recalling displays (36,37) and making impulse purchases (37) but no short-term change in tobacco use prevalence among adults or adolescents (36), although mathematical models suggest that these policies would reduce tobacco use over time (38). Multiple online resources provide lists of policy options together with summaries of and citations to evidence in support of their impact (Table).

### Data spring 7: Amount of political will

Data on political will include data on attitudes, beliefs, and vested interest among community members and key political decision makers in POS marketing relative to other priorities (eg, maintaining a business-friendly climate) and toward POS marketing policy solutions. Information on public support for policies can be gathered through public opinion polls or focus groups and interviews (38,39). Resources for conducting public opinion surveys, such as those provided by CounterTobacco.org, guide communities through the process and help leverage results to persuade policy makers (http://countertobacco.org/resources-tools/public-opinion-surveys).

Community members’ political will is central to getting an issue on the agenda of decision-making bodies such as town councils. Community members must also have the will and ability to anticipate and prepare for industry interference in policy activities (Table). The political will of decision makers (eg, town council members, mayors) also is important. Key decision makers need to view retail tobacco policy as a pressing issue so that they will propose policy changes and support their enactment. One-on-one meetings are one strategy for understanding local decision makers’ views of tobacco retail marketing policies in general and their positions on specific strategies.

## Toward Policy Change: Merging Data Springs to Activate Policy Streams

We outlined how community partnerships can draw on the 7 data springs to activate the 3 streams central to Kingdon’s theory of successful policy change. Springs 1, 2, and 3 can be tapped into to identify the local problem (Kingdon’s first stream). Engaging community members in collecting and sharing these data are a powerful tool for building political will to work for change (Kingdon’s third stream). Springs 1, 2, and 3 also contribute to policy formulation (Kingdon’s second stream) by providing local data on the problem, data that are key to selecting and targeting policy strategies to align with local needs. Springs 4 through 7 further contribute to Kingdon’s second stream by providing the data needed to stage policy strategies to fit local readiness for policy change. Spring 7 also provides data suggested to design a policy campaign to generate the political and community will needed to enact new policy (Kingdon’s third stream).

We identified 7 “springs of evidence” that are essential to local public health officials’ work to formulate the POS tobacco policies with the greatest potential to decrease the burden of tobacco use in their communities. We acknowledge that some data springs do not exist or are shallow (eg, many states do not require tobacco retailer licensing, and policy contexts are difficult to track systematically), and we encourage continued investment in data collection and sharing. To the extent possible, we also encourage policy effectiveness studies to further inform policy development.

Our model focuses on tobacco retail policy, but the conceptual data springs are applicable to other health-supporting policy initiatives. Although the sources of data may differ, and new springs may need to be added, the conceptual process of evidence-informed decision making can be applied to a range of retail policies (food, alcohol, physical activity environment). Harnessing relevant data to change local policy is key to creating health-supporting community environments and improving population health.
